# Diagnostic performances of Exacto® Triplex rapid test for diagnosis of HIV/HCV/HBsAg: a multicenter, cross-sectional, field study in the Central African Republic

**DOI:** 10.11604/pamj.2022.43.21.36041

**Published:** 2022-09-14

**Authors:** Christian Diamant Mossoro-Kpinde, Ginette Claude Mireille Kalla, Coretha Baguida-Bokia, Simplice Sombot-Ndicki, Christelle Bobossi, Serge Tonen-Wolyec, François-Xavier Mbopi-Kéou, Laurent Bélec

**Affiliations:** 1 Laboratoire National de Biologie Clinique et de Santé Publique, Bangui, République Centrafricaine,; 2 Faculté des Sciences de la Santé, Université de Bangui, Bangui, République Centrafricaine,; 3 Faculty of Medicine and Biomedical Science, University of Yaounde I, Yaounde, Cameroon,; 4 Faculté de Médecine et de Pharmacie, Université de Kisangani, Kisangani, République Démocratique du Congo,; 5 Laboratoire de Virologie, Hôpital Européen Georges Pompidou, Assistance Publique-Hôpitaux de Paris (AP-HP) et Université Paris Cité, Paris, France

**Keywords:** Human immunodeficiency virus, hepatitis C virus, hepatitis B virus, multiplex immunochromatographic rapid test, diagnostic performances, Central Africa

## Abstract

**Introduction:**

the Exacto® Triplex HIV/HCV/HBsAg (Biosynex, Strasbourg, France) consists in lateral flow, immunochromatographic rapid diagnostic test simultaneously detecting human immunodeficiency virus (HIV)-1 and HIV-2 and hepatitis C virus (HCV)- specific antibodies (IgG and IgM) and hepatitis B virus (HBV) surface antigen (HBsAg) in serum, plasma and whole blood. We herein evaluated its diagnostic performances in the Central African Republic (CAR).

**Methods:**

cross-sectional study was conducted on prospectively collected panel of 550 sera from adult inpatients living in Bangui, including 200 HIV-positive, 100 HBsAg-positive, 50 HCV-positive, 200 negatives to three viruses according to reference immuno-enzymatic serological tests including Murex HCV (Diasorin, Saluggia, Italy) for HCV, Murex HBsAg (Diasorin) for HBV, Genscreen ULTRA HIV Ag-Ab HIV-1/2 Version 2 (Bio-Rad, Marnes-la-Coquette, France) and Murex HIV 1.2.0 Ag/Ab Combination (Diasorin), the 2 tests associated in the parallel algorithm for the reference strategy for the diagnosis of HIV in CAR. Serum samples were tested blindly in duplicate. The findings are reported following the Strengthening the Reporting of Observational Studies in Epidemiology (STROBE) guidelines.

**Results:**

the Exacto® Triplex showed 99.5% (95% CI; 98.5-100.0), 96.0% [90.6-100.0] and 99.0% [97.1-100.0] sensitivities for HIV, HCV and HBsAg, respectively. The specificity, positive and negative predictive values (PPV and NPV) were 100.0% for all three viruses. The Youden's J index and Cohen's κ coefficient were 0.99 for HIV and HBsAg. For HCV, Youden's J and Cohen's κ coefficient were 0.96 and 0.98, respectively. In the epidemiological context of the CAR, the PPV and NPV for all three viral infections were high (≥99.0% to 100%).

**Conclusion:**

taken together, our STROBE-compliant study demonstrates that the Exacto® Triplex HIV/HCV/HBsAg showed high sensitivity and specificity for HIV and HBsAg (≥99.0%), and relatively high sensitivity (96.0%) and high specificity (100%) for HCV. These analytical performances are within the limits required by the WHO (i.e. sensitivity ≥99.0% and specificity ≥98.0%) for HIV and HBV. The Exacto® Triplex HIV/HCV/HBsAg is user-friendly at low cost, and appears highly desirable for routine use in the CAR, and likely other Central African countries.

## Introduction

The Exacto® Triplex HIV/HCV/HBsAg (Biosynex, Strasbourg, France) consists in manually performed, visually interpreted, lateral flow, immunochromatographic rapid diagnostic test simultaneously detecting in 15 minutes human immunodeficiency virus (HIV)-1 and HIV-2 and hepatitis C virus (HCV)- specific antibodies (IgG and IgM) and hepatitis B virus (HBV) surface antigen (HBsAg) in serum, plasma and whole blood [1]. The Exacto® Triplex HIV/HCV/HBsAg allows to diagnose rapidly three viral infections sharing common risk factors, but which are generally diagnosed with distinct assays [2-4]. The interest of such a rapid test is to be able to diagnose easily three viral infections with the same blood matrix, most often capillary blood, at a lower cost and without waste [1].

The choice of a reliable serological tests is based primarily on the reliability of the results of their comparative evaluation to the reference tests. According to the World Health Organization (WHO), the evaluation of serological tests for HIV as well as HBV and HCV is part of a continuous process comprising three phases, including laboratory evaluation (phase I) to assess the diagnostic performances such as sensitivity and specificity, field evaluation (phase II) and further assessment of the performances with time (phase III) [4-6]. It is important that the evaluation of serological tests is carried out in real life, i.e. under the local conditions of actual use [5,6]. Indeed, even if a test has been pre-qualified by the WHO or a competent organization, it may show to be insufficiently efficient in field conditions. The only one previous virological evaluation of the Exacto® Triplex HIV/HCV/HBsAg was carried out in France using sera from people living in France and from HIV-2-infected patients living in West Africa and showed acceptable diagnostic performances for routine use [1], but the authors mentioned the need for future evaluations in the field, especially in limited resources settings. For example, due to the high genetic diversity of HIV in most African countries, some screening tests have been shown to be less sensitive in detecting certain variants of HIV-1 [7].

We herein evaluated the diagnostic performances of the Exacto® Triplex HIV/HCV/HBsAg in the Central African Republic (CAR), a country with limited resources of more than 5 million inhabitants with high prevalence of HIV, HCV and HBV of around 3.7%, 4.7% and 15.5%, respectively [8-10]. The interest of carrying out this study in the CAR is major for at least three following reasons: i) The diagnostic performances of the Exacto® Triplex HIV/HCV/HBsAg have never been reported in African country; ii) HIV strains in the CAR are characterized by a broad genetic diversity with a great risk of modifying the diagnostic performances of rapid tests [7,11]; (iii) The risk of inconclusive sera with unspecific reactivity is high for HIV rapid tests in the Central African setting [12].

## Methods

**Study design:** this cross-sectional study was designed to assess the diagnostic performances of the Exacto® Triplex from a panel of serum samples prospectively collected for HIV, HCV and HBV seroprevalence surveys between November and December 2020 at the two main reference laboratories of the CAR, including the *Laboratoire National de Biologie Clinique et de Santé Publique* of Bangui, the capital city of the CAR, and the *Institut Pasteur de Bangui*.

**Reference serological assays:** the reference serological assays to assess the reference serum panel were CE-marked ELISA tests available in the CAR. For HIV serology, the reference serological strategy was that previously validated at the national level by the *Laboratoire National de Biologie Clinique et de Santé Publique*, Bangui, including the parallel screening by Genscreen ULTRA HIV Ag-Ab HIV-1/2 Version 2 (Bio-Rad, Marnes-la-Coquette, France) and Murex HIV 1.2.0 Ag/Ab Combination (Diasorin, Saluggia, Italy); confirmation of positive samples was carried out by a third available HIV diagnostic test, as recommended by WHO [6] and previously described [13]. For HBV and HCV infections, the reference assays were the Murex HBsAg Murex HCV from Diasorin (Saluggia, Italy). Serum samples were tested blindly in duplicate. Test cut-offs for positivity, negativity and doubtful zone were those defined in the instructions for use by the manufacturers of the reference ELISA assays. Sera with doubtful results with ELISA were not included in the reference panel, as recommended by the WHO [5].

**Exacto® Triplex HIV/HCV/HBsAg:** the Exacto® Triplex HIV/HCV/HBsAg consists of manually performed, visually interpreted, qualitative, in vitro lateral flow immunoassays for simultaneous detection of HIV, and HCV-specific antibodies (Ab; IgG and IgM) and HBsAg in human whole blood (venipuncture or fingerstick), serum, or plasma. The test uses synthetic antigens (gp41, gp36) able to detect antibodies against HIV-1 or HIV-2, monoclonal antibody to detect HBsAg, and fusion recombinant multiepitope chimeric HCV protein containing structural (core) and nonstructural (NS3, NS4, and NS5) HCV antigens to detect antibodies against HCV, which are all bound to the solid phase membrane. The Exacto® Triplex HIV/HCV/HBsAg was carried out according to the instructions for use of the manufacturer. The quantity of the whole blood sample and serum or plasma needed to perform the test is 50 μL and 25 μL, respectively. The time of migration is 15 minutes. The Exacto® Triplex HIV/HCV/HBsAg is packaged individually containing all of the necessary components to carry out the test. The price of the Exacto® Triplex HIV/HCV/HBsAg is around 5 US $.

**Reference serum panel and further testing:** the reference panel was constituted by serum samples prospectively collected for HIV, HCV and HBV seroprevalence surveys. For HIV infection, the sample size was 200 HIV-positive and 200 HIV-negative sera, according to the WHO recommendations for phase I evaluation [5]. All sera co-infected by HIV and HBV or HIV and HCV were excluded. For HBV infection, the sample size was arbitrarily fixed to 100 HIV- and HCV- negative and HBsAg-positive sera. For HCV infection, the sample size was arbitrarily fixed to 50 HIV- and HBV- negative and HCV-positive sera. Note that the infectivity of HCV-specific antibodies positive sera could not be assessed, because molecular detection of HCV RNA was not available. All sera were kept frozen at -80°C until processing. The reference serum panel was further tested with the Exacto® Triplex HIV/HCV/HBsAg by two clinical microbiologists blinded regarding the sample groups. The results categories of the Exacto® Triplex HIV/HCV/HBsAg were negative, positive or indeterminate with a doubtful band. Indeterminate, doubtful readings were further read by a third microbiologist. In this study, the clinical information of the sample source participants was not known. The performers/readers also did not know the results of the samples analysed by reference tests in order to avoid any classification bias.

**Inclusion criteria:** they were as follows: age more than 18 years, signed informed consent, availability of simple demographic data on participants (e.g., age and gender), availability of aliquoted serum, availability of serological results for HIV, HBsAg and HCV serologies by reference ELISA assays.

**Exclusion criteria:** they were as follows: refusal to participate in the study, inconsistent demographic information, partial serological results, sera with indeterminate results by reference ELISA assays, sera positive for HIV and HBV or HIV and HCV or for HIV, HBV and HCV.

**Statistical analysis:** data were entered into an Excel database. The results were presented along with their two-sided 95% confidence interval (CI) using the Wilson score bounds for categorical variable [14]. The results of HIV-specific antibodies, HBsAg and HCV-specific antibodies detection by the ELISA assays were used as the reference standard to estimate the sensitivity and specificity of the Exacto® Triplex HIV/HCV/HBsAg, with corresponding 95% CI. The reliability between the Exacto® Triplex HIV/HCV/HBsAg and the comparator assays was estimated by Cohen´s κ coefficient [15], and the degree of agreement was determined as ranked by Landlis and Koch [16], as follows: < 0 as indicating no agreement, 0-0.20 as slight, 0.21-0.40 as fair, 0.41-0.60 as moderate, 0.61-0.80 as substantial, and 0.81-1 as almost perfect agreement. The accuracy of the Exacto® Triplex HIV/HCV/HBsAg to correctly detect HIV-specific antibodies, HBsAg and HCV-specific antibodies was estimated by Youden´s J index (J = sensitivity + specificity - 1) [17]. Positive predictive value (PPV) and negative predictive value (NPV) were calculated for the Exacto® Triplex HIV/HCV/HBsAg according to the Bayes´ formula, using the overall prevalence of HIV of 3.7%, the HCV seroprevalence of 4.7% and the HBsAg prevalence of 15.5% in the CAR [8-10].

**Ethical approval:** this study, which belongs to the collaboration within the framework of the inter-hospital or inter-laboratory exchange program so-called “*ESTHER” (“Ensemble pour une Solidarité Thérapeutique Hospitalière en Réseau*”), between France and the Central African Republic, in the field of infectious diseases, in particular HIV infection and viral hepatitis, was formally approved by the Scientific Committee of the Faculté des Sciences de la Santé in Bangui, constituting the National Ethical Committee (Reference #2UB/FACSS/CSVPR/59). Written informed consent was obtained from all participants for their anonymized information to be published in this article.

## Results

**Reference panel:** the reference panel of 550 serum samples prospectively collected for HIV, HCV and HBV seroprevalence surveys included 200 HIV-positive, 100 HBsAg-positive, 50 HCV-positive and 200 negative to both 3 viruses. The mean age of participants was 35.7 years (range, 18-64 years); and 237 (52.7%) were female.

**Testing of the reference panel by Exacto® Triplex HIV/HCV/HBsAg:** among the 200 serum samples positive by the HIV reference algorithm, one was negative by Exacto® Triplex HIV/HCV/HBsAg. One of the 100 HBsAg-positive serum samples by reference serology was negative by Exacto® Triplex HIV/HCV/HBsAg. For HCV, negative results were observed with Exacto® Triplex HIV/HCV/HBsAg for 2 samples out of the 50 sera positive by reference serology. The two negative samples for HCV with the Exacto® Triplex HIV/HCV/HBsAg had low optical densities (OD) in HCV ELISA, with low OD/Cut-off ratios of 1.51 and 1.98, respectively. The 200 HIV-, HBsAg- and HCV- negative sera were also negative by the Exacto® Triplex HIV/HCV/HBsAg.

The HIV, HBsAg and HCV bands intensities on Exacto® Triplex HIV/HCV/HBsAg strips were proportional to the level of the final optical densities observed for all three reference ELISA assays (not shown). The majority of sera samples gave marked intensity bands on Exacto® Triplex HIV/HCV/HBsAg, except for about 10% whose intensity bands were moderate or weak (not shown). The diagnostic performances of the Exacto® Triplex HIV/HCV/HBsAg are shown in the Table 1. For HIV infection and chronic HBV infection, the sensitivities and the specificities of Exacto® Triplex were ≥99.0% and 100%, respectively. For HCV infection of unknown status, the sensitivity and the specificity of the Exacto® Triplex were 96.0% and 100%, respectively. For all three viral infections, the Exacto® Triplex showed high accuracy (Youden´s J index) and agreement (Cohen´s κ coefficient). In the epidemiological context of the CAR, the PPV and NPV for all three viral infections were high (≥99.0% to 100%) (Table 1).

**Table 1:** diagnostic performances of Exacto® Triplex HIV/HCV/HBsAg, expressed in percentage, by comparison with reference immune-enzymatic assays available in the Central African Republic, including in parallel Genscreen ULTRA HIV Ag-Ab HIV-1/2 Version 2, (Bio-Rad) and Murex HIV 1.2.0 Ag/Ab Combination (Diasorin) for HIV infection, Murex HBsAg (Diasorin) for HBsAg, and Murex HCV (Diasorin) for HCV infection

Viral markers	Type of infection	Sensitivity [95% CI]^£^	Specificity [95% CI]^£^	Positive predictive value* [95% CI]^£^	Negative predictive value* [95% CI]^£^	Index J of Youden [Se+Sp-1]	Coefficient κ of Cohen
**Anti-HIV Ab**	Chronic	99.5 [98.5-100.0]	100.0 [99.9-100.0]	100.0 [99.9-100.0]	99.9 [99.0-100.0]	0.99	0.99
**Anti-HCV Ab**	Unknown	96.0 [90.6-100.0]	100.0 [99.9-100.0]	100.0 [99.9-100.0]	99.7 [98.3-99.9]	0.96	0.98
**HBsAg**	Chronic	99.0 [97.1-100.0]	100.0 [99.9-100.0]	100.0 [99.9-100.0]	99.8 [98.4-99.9]	0.99	0.99

* The positive predictive values and negative predictive values were calculated by using Bayes' formula taking account of the HIV prevalence of 3.7%, the HCV seroprevalence of 4.7% and the HBsAg prevalence of 15.5% in the Central Africa Republic. ^£^The results were presented as a 95% CI using the Wilson score bounds. Ag: antigen; Ab: antibodies; CI: confidence interval; Se: sensitivity; Sp: specificity

## Discussion

We herein evaluated the diagnostic performances of the Exacto® Triplex HIV/HCV/HBsAg in the field in the CAR. This study constitutes to our knowledge the first phase I evaluation of the Exacto® Triplex HIV/HCV/HBsAg in sub-Saharan Africa in a context of resource-constrained setting. Overall, the performances of the Exacto® Triplex were high for HIV and chronic HBV infection, good for HCV of unknown status, and of the same order than those previously reported by Robin and colleagues in Paris [1]. In particular, the Exacto® Triplex HIV/HCV/HBsAg fulfilled the WHO recommendations for HIV rapid diagnostic tests of a sensitivity >99.0% and a specificity >98.0% to be valid [18]. This observation is remarkable by it-self for a HIV rapid test, as sera from Central Africa are considered “difficult”, often giving rise to non-specific reactivities [12,19,20]. Indeed, the prevalence of so-called “inconclusive” sera is particularly high in certain regions of sub-Saharan Africa, such as in Central Africa [20]. Many infectious diseases can cause nonspecific polyclonal reactivities or circulating immune complexes that can lead to false positive results with HIV screening tests [21]. Among the rapid screening tests, immunochromatography tests risk “catching” circulating immune complexes easily, that could be the source of false positive results [20-22]. The relatively high prevalence of false negative results for HCV (4%) may have resulted from decreased level of HCV-specific antibodies and/or very weak affinity of HCV-specific antibodies for their antigens in case of spontaneously cured HCV infection [23].

The Exacto® Triplex has several advantages. The test does not require highly trained personnel or any special equipment for the whole blood. In addition to serum and plasma, capillary blood, very easy to collect by finger prick, may be used [1]. Reading of the test results is visual. Taken together, our observations indicate that the Exacto® Triplex HIV/HCV/HBsAg is suitable for screening HIV, HCV, HBV (HBsAg) in the CAR, a country where HIV strains of very high genetic diversity circulate and which is strongly affected by these 3 viral infections, in order to improve the “cascade of screening” and quite possibly linkage-to-care with reduced cost [11,24-26]. More generally, the Exacto® Triplex seems particularly adapted to low-income African countries, in particular for mass screening for HIV, HCV and HBV in sub-Saharan Africa [24], in patients attending sexually transmitted infection clinic [25], in childbearing-aged women [26], and likely in blood transfusion screening. In addition, a recent pilot study carried out in the Democratic Republic of the Congo has reported evidence for the first time in sub-Saharan Africa on good practicability and high acceptability of a prototype Triplex HIV/HCV/HBsAg self-test, providing the rational basis of using self-test harboring four bands of interest, i.e. the control, HIV, HCV, and HBsAg bands [27].

**Study limitations:** the study has some limitations. Firstly, this study was conducted on serum samples and not capillary blood. Secondly, the sample size of included samples was consistent with WHO recommendations for HIV infection [5], but was arbitrarily chosen for HBV (HBsAg) and HCV infections. In particular, the number of HCV positive samples was low, and the performance of Exacto® Triplex HIV/HCV/HBsAg obtained in this study for HCV deserves certain reservation and may require confirmation on a much larger sample size. Thirdly, the virological status of serum samples positive for HCV-specific antibodies was not assessed by molecular biology. Fourthly, the exclusion of sera with indeterminate results by reference ELISA assays, and of sera positive for HIV and HBV or HIV and HCV or eventually for HIV, HBV and HCV, could have introduced a selection bias. Fifthly, the evaluation of the specificity of Exacto® Triplex HIV/HCV/HBsAg only included negative samples for the 3 viruses. The fact of not having used positive samples for viruses different from the viruses whose specificity of the test was evaluated could have constituted a limitation of the study. However, the WHO guidelines for the evaluation of tests, particularly those for HIV, only indicate about positive and negative samples for the indicated virus, without specifying any virus different from that or those concerned by the evaluation [4-6]. Finally, our study in the CAR should be completed by further evaluations in the field, especially in other sub-Saharan settings endemic from pathogens known to interfere with testing such as malaria and other infectious diseases at the origin of polyclonal B cell activation [12,20,21].

## Conclusion

The Exacto® Triplex HIV/HCV/HBsAg showed high sensitivity and specificity for HIV and HBsAg (≥99.0%), and relatively high sensitivity (96.0%) and high specificity (100%) for HCV. Taken together, these diagnostic performances associated with the easy use and low cost of the Exacto® Triplex HIV/HCV/HBsAg make this rapid test highly desirable for routine use in the CAR, and likely other Central African countries.

### What is known about this topic

- Multiplex rapid tests for serological diagnosis of HIV, HCV, HBV may improve the linkage-to-care with reduced cost in sub-Saharan Africa which is strongly affected by these 3 viral infections;
- Field validation of the Exacto® Triplex HIV/HCV/HBsAg constitutes a mandatory prerequisite before use, and has never been reported in Central Africa;
- Rapid tests for serological diagnosis of HIV, HCV, HBV may lack of specificity in Central Africa.

### What this study adds

- Exacto® Triplex HIV/HCV/HBsAg is highly sensitive and specific for HIV and HBsAg (≥99.0%), and relatively highly sensitive (96.0%) and specificity (100%) for HCV;
- Exacto® Triplex HIV/HCV/HBsAg can be used safely with high confidence in Central Africa.
